# Understanding the squamous cell carcinoma immune microenvironment

**DOI:** 10.3389/fimmu.2023.1084873

**Published:** 2023-01-30

**Authors:** Vahide Saeidi, Nicole Doudican, John A. Carucci

**Affiliations:** Section of Dermatologic Surgery, Ronald O. Perelman Department of Dermatology, New York University Langone Medical Center, New York, NY, United States

**Keywords:** squamous cell carcinoma, tumor microenvironment, PD-1, tumor infiltrating lymphocytes, exhausted T cells, cytokines

## Abstract

Primary cutaneous squamous cell carcinoma (cSCC) is the second most common human cancer with a rising incidence of about 1.8 million in the United States annually. Primary cSCC is usually curable by surgery; however, in some cases, cSCC eventuates in nodal metastasis and death from disease specific death. cSCC results in up to 15,000 deaths each year in the United States. Until recently, non-surgical options for treatment of locally advanced or metastatic cSCC were largely ineffective. With the advent of checkpoint inhibitor immunotherapy, including cemiplimab and pembrolizumab, response rates climbed to 50%, representing a vast improvement over chemotherapeutic agents used previously. Herein, we discuss the phenotype and function of SCC associated Langerhans cells, dendritic cells, macrophages, myeloid derived suppressor cells and T cells as well as SCC-associated lymphatics and blood vessels. Possible role(s) of SCC-associated cytokines in progression and invasion are reviewed. We also discuss the SCC immune microenvironment in the context of currently available and pipeline therapeutics.

## 1 Introduction

Cutaneous SCC (cSCC) is the second most frequent skin cancer in the United States (US) with 1.8 million new cases each year, and its global incidence rate has been reported to increase 3-7% annually (1, 2). cSCC lesions appear in regions that are most exposed to ultraviolet (UV); the head and the neck are the most common sites followed by the trunk and extremities (3).

UV radiation can alter the genome of epidermal cells and cause SCC development and subsequent metastasis, usually to nearby lymph nodes. A complex network of genes (TP53, CDKN2A, NOTCH1, NOTCH2, EGFR and TERT) and molecular pathways (RAS/RAF/MEK/ERK and PI3K/AKT/mTOR) are associated with the pathogenesis of cSCC (4). Also, recent findings identified EP300, PBRM1, USP28, and CHUK as four novel genes that are mutated in greater than 10% of cSCCs (5). The top three recurrently altered genes in metastatic cSCCs are TP53, CDKN2A, and NOTCH1/2 (6–8).

In addition to UV exposure ionizing radiation, fair skin, chronic immunosuppression, genetic conditions, the presence of chronic wounds or scars, smoking, chemical carcinogens, and human papillomavirus (HPV) infection are the other risk factors of cSCC development (9). The vast majority of cSCC cases are treated successfully by excision with clear margins (10, 11); however, these tumors can be aggressive and responsible for most of the ~15,000 non-melanoma skin cancer deaths in the United States each year (1). Patients with localized cSCC have a favorable prognosis with a 5-year survival rate of 99% following Mohs micrographic surgery (12, 13). Metastasis affects approximately 3.7%-5.2% of all SCC patients (14). The expected 5-year and 10-year survival rates in these patients decreases to 25-50% and 16%, respectively (11, 15–17).

**Figure 1:**
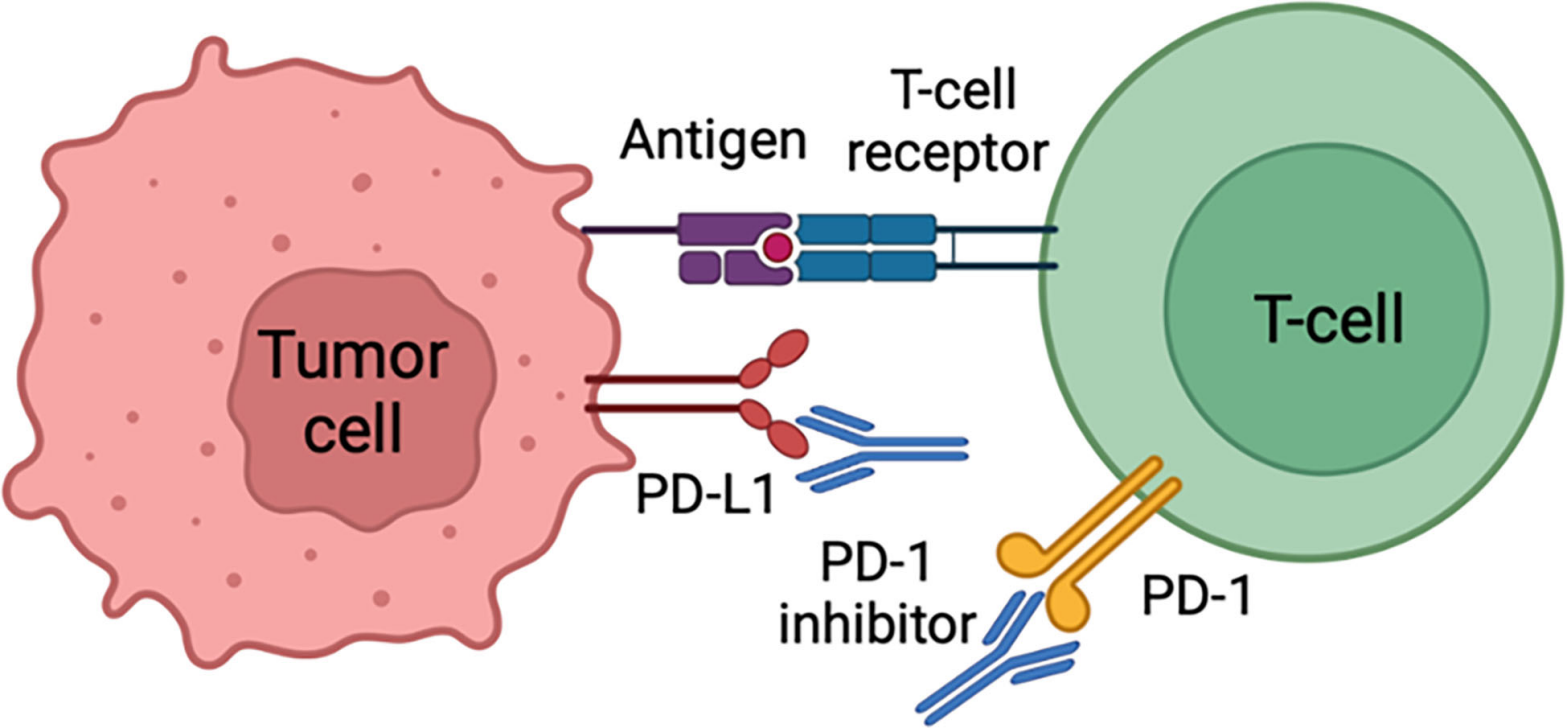
Cancer cells can evade immune surveillance by expressing PD-L1 protein that acts as a “stop sign” to inactivate T cells. PD-L1 attaches to PD-1 and B7.1 T cell receptors, both of which inactivate T cells. Cemiplimab prevents T cell inactivation and subsequently increases anti-cancer activity through PD-L1 blockade.

Advanced cSCC is described as either a locally advanced disease that is untreatable by surgery or radiation therapy (RT), a metastatic disease with distant metastases, or large, multiple, and extracapsular nodal disease with a high risk of recurrence despite lymphadenectomy and radiation therapy (18). Cemiplimab, an immune checkpoint inhibitor, is the first medication approved in the United States for advanced cSCC (19). It is a human monoclonal antibody that inhibits the PD-1 pathway by blocking T-cell inactivation, thus assisting the immune system in fighting cancer cells (20) as illustrated in **Figure 1**. Cemiplimab exhibits an overall response rate of 50%, which is a significant improvement over conventional chemotherapy. It has been shown that cemiplimab has a significant antitumor function with long-lasting response, and acceptable safety profile in patients (19). Pembrolizumab is another PD-1 inhibitor, with a similar mechanism to cemiplimab, and has been recently approved in the United States for recurrent or metastatic cSCC that is uncurable with surgery or radiation therapy (21). A case of metastatic cSCC treated with nivolumab, another PD-1 inhibitor, has been reported, and the patient exhibited a complete response to this treatment (22). In another case report, a patient with unresectable recurrent scalp cSCC with meningeal invasion was successfully treated with nivolumab monotherapy (23).

Lymphocyte activation gene 3 (LAG3) is an inhibitory receptor that is expressed on CD4^+^, CD8^+^, regulatory T (T-reg) cell, natural killer cell, B cell, and other immune cells (24). LAG3 serves a negative regulatory role in cancer immunology by interacting with its ligands. Higher LAG3 expression has been reported in head and neck squamous cell carcinoma compared to normal tissues. Therefore, LAG3-targeting agents could represent another promising checkpoint inhibitor immunotherapy for these malignancies (25). Combining immunotherapy and radiotherapy is another cutting-edge method of treating cSCC (26). The trials of radiation therapy and cemiplimab in patients with skin cancer (NCT05574101) as well as radiotherapy in combination with atezolizumab (PD-L1 inhibitor) in locally advanced borderline resectable or unresectable cSCC (NCT05085496) are ongoing. Another ongoing trial is testing cetuximab (EGFR inhibitor) before surgery in the treatment of patients with aggressive locally advanced skin cancer (NCT02324608).

The efficacy of talimogene laherparepvec (oncolytic viral immunotherapy) and panitumumab (EGFR inhibitor) for the treatment of locally advanced or metastatic cSCC is being researched in another ongoing trial (NCT04163952).

The development and progression of non-melanoma skin cancer (NMSC) are significantly influenced by immune system function (27). An increased incidence of cSCC in immunocompromised solid organ transplant recipients indicates the critical role of the immune surveillance in host protection (28). The immune system recognizes cancer cells as abnormal and can eliminate them in some cases (29); however, tumor cells might evade immune surveillance through immunoediting processes (30). Cancer cells utilize several mechanisms to escape immune surveillance, including MHC loss and expression of immunosuppressive factors, such as IL-6, IL-10, TGF-β, prostaglandins, and Fas ligand (31, 32).

The tumor microenvironment is characterized as a combination of tumoral and non-tumoral cells at the dynamic interface of neoplasia (33). Although non-tumoral cells within the tumor microenvironment may have protective functions in limiting tumor progression, many studies show that they have also an important role in tumor growth and metastasis (34). Therefore, it is crucial to understand the features of the cSCC tumor-associated immune microenvironment in detail to develop reliable prognostic markers and new advanced treatments.

In this review, phenotype and functions of cSCC-associated Langerhans cells, dendritic cells, macrophages, myeloid-derived suppressor cells and T cells as well as cSCC-associated lymphatics and blood vessels are discussed. Moreover, the potential roles of cSCC-associated cytokines in progression and invasion of the tumor are described.

## 2 Myeloid-derived suppressor cells in SCC

Myeloid-derived suppressor cells (MDSCs) are pathologically activated neutrophils and monocytes with immunosuppressive activity. They participate in the regulation of immune responses in many pathological conditions, such as cancer, chronic infection, sepsis, and autoimmunity. Two major groups of MDSCs in humans include granulocytic/polymorphonuclear MDSCs (PMN-MDSCs) and monocytic MDSCs (M-MDSCs), which originate from the granulocytic and monocytic myeloid cell lineages, respectively (35). MDSCs are related to poor outcomes in cancer (36). It has been shown that high levels of circulating MDSC in patients with solid tumors, were related to poor overall survival (37).

In cancer patients, these cells express the common myeloid marker CD33 but not mature myeloid and lymphoid cell markers in cancer patients. In humans, MDSCs are identifiable as lineage (CD3, CD14, CD19, CD56)–negative, HLA-DR–negative, and CD33-positive or CD33^+^CD14^- ^CD11b^+^ cells (38, 39).

The signals driving MDSCs development occur in two partially overlapping phases. Expansion of immature myeloid cells occurs in phase 1, and neutrophils and monocytes convert to pathologically activated MDSCs in phase 2 (38).

MDSCs are one of the major factors responsible for immune suppression in cancers that not only cause tumor progression but also result in the failure of immunotherapy (39). Arginase, nitric oxide (NO), and reactive oxygen species (ROS) have all been shown to play a role in MDSC-mediated T-cell suppression (40). MDSCs are critical producers of NO in SCC, which suppresses E-selectin expression on tumor vessels. Subsequently, the entry of skin homing T-cells into tumors are restricted, resulting in evasion of SCC from immune detection (41).

Clearly, a successful cancer immunotherapy will be possible if the immune suppressive factors can be eliminated from the body. As MDSCs are one of the major immune suppressive factors in cancers, the challenge of effectively and selectively targeting MDSCs remains (39). Medications that diminish NO production e.g., iNOS inhibitors, may be effective in the treatment of SCCs and their premalignant precursor lesions actinic keratoses through improvement of anti-tumor immune responses (41). Based on earlier studies, all-trans retinoic acid (ATRA) promotes the differentiation of M-MDSCs into macrophages and DCs and apoptosis of PMN-MDSCs in both mice and humans (42–44). Concurrent use of ATRA therapy with CTLA-4 blockade was tested in melanoma patients and resulted in decrease in the number of circulating MDSCs. Therefore, targeting MDSCs in combination with immunotherapies may improve response rates and effectiveness in other skin cancers (45).

## 3 Tumor-associated macrophages

Macrophages are important tumor-infiltrating cells (46) contributing to different carcinogenesis stages, including initiation, growth, invasion, and metastasis (47, 48). More macrophages are present in SCC compared with normal skin (49). Macrophages surrounding and penetrating the tumor are termed tumor-associated macrophages (TAMS) (46).

In response to tumors, macrophages display a polarized reaction defined by two different states: classically activated macrophage (M1) and alternatively activated macrophage (M2). M1 macrophages are activated by interferon-γ (IFN-γ), bacterial lipopolysaccharide (LPS), or tumor necrosis factor-α (TNF-α) and release interleukin 12 (IL-12) to prevent tumor growth. In contrast, M2 macrophages are activated by IL-4 and release IL-10, which contributes to tumor progression (27, 50–52).

**Figure 2:**
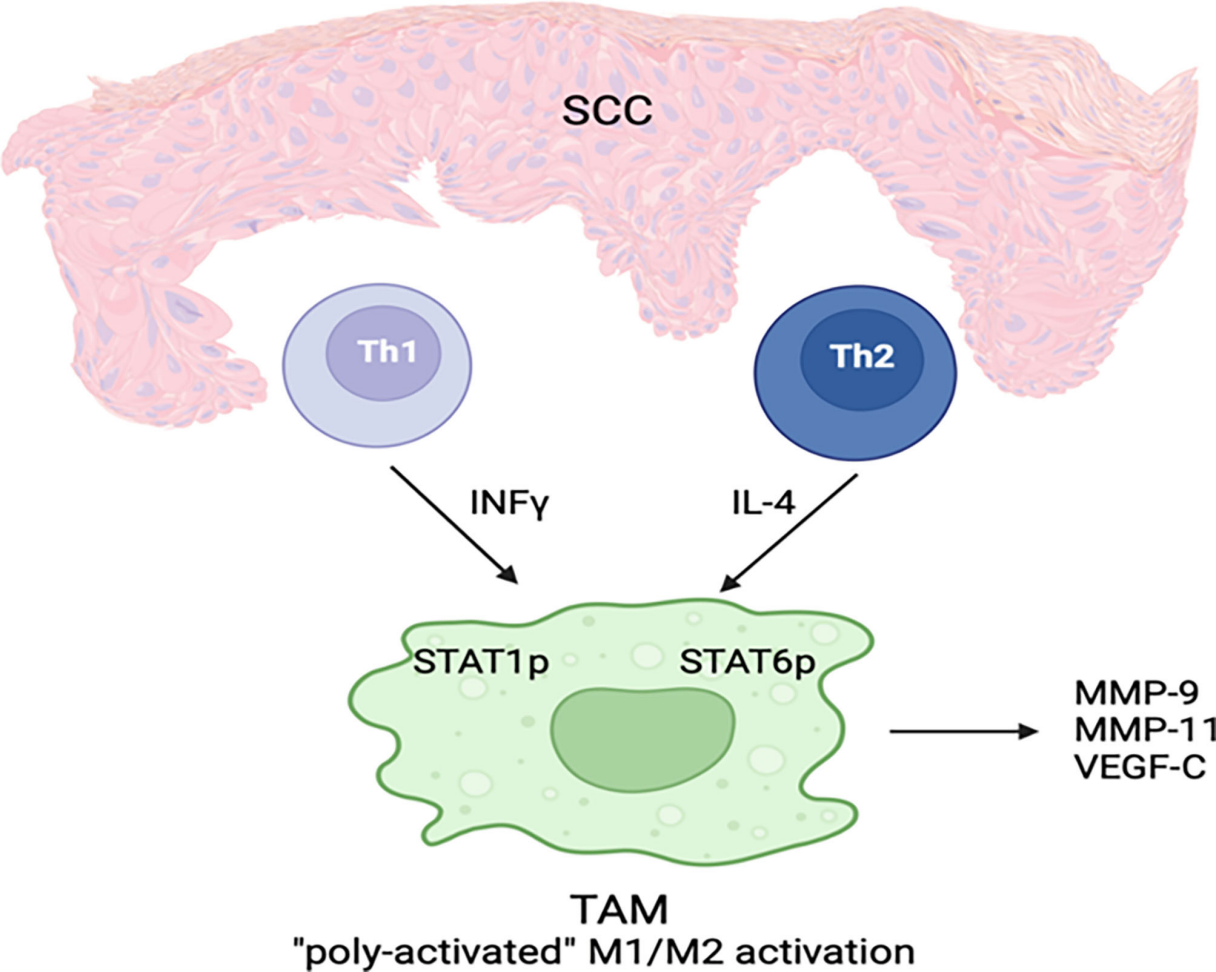
A subset of TAMs in cSCC displays both classical and alternative activation features simultaneously. IFN-γ and IL-4 are secreted by Th1 and Th2 cells, respectively, in the cSCC microenvironment. As a result of these cytokines, which activate M1 classic and M2 alternate phenotypes, poly-activated TAMs are generated. STAT1 and STAT6 phosphorylation as well as MMP-9, MMP-11, and VEGF-C expression are characteristic features of TAMs.

Tumor-associated macrophages have many similar characteristics to alternatively activated macrophages (M2 macrophages) (46). Based on recent studies, macrophage activation in SCC is heterogenous and there are three types of TAMs: TAMs expressing M1 markers, TAMs expressing M2 markers and TAMs simultaneously expressing M1 and M2 (49) (**Figure 2**). It is believed that tumors can generate a dynamic microenvironment that alters the TAMs into macrophages that help tumor growth (53). Weaker classical macrophage activation in SCC cause TAMs to produce more tumorigenic growth factors (49). Increased TAM levels are associated with poor prognosis in various human malignancies (47, 48, 54).

Heterogeneous activation of TAMs in SCC suggests potential treatment strategies contributing to the induction of a more dominant M1 activation state with anti-cancer phenotype (27).

TAMs in SCC may produce matrix metalloproteinases (MMPs) that may aid tumor invasion. A positive correlation between MMP-9 (gelatinase B) and MMP-11 (stromelysin-3) proteins and increased tumor aggressiveness has been revealed (55–58). TAMs also contribute to lymphangiogenesis through vascular endothelial growth factor-C (VEGF-C) expression (59). It has been reported that enhanced lymph vessel density is related to increased risk of metastasis in the oral cavity SCC and melanoma (60, 61).

TAM densities and functional immunophenotypes differ in human cutaneous SCCs and BCCs, which can contribute to behavioral differences between these two tumors. It has been shown that SCCs express more TAM-associated markers (MMP-9, arginase-1, CD127 and CD40) compared with BCCs, and TAMs in SCC have a higher density and polarization state. Lactic acid levels are higher in SCCs compared with BCCs, and tumor-derived lactic acid is an important factor playing a role in TAM polarization in SCCs (62).

In fact, TAMs in SCC, due to weaker classical macrophage activation and higher production of tumorigenic growth factors, are unable to prevent tumor genesis and in fact they can even facilitate tumor growth; however, they contribute to tumor invasion and metastasis through production of high levels of MMPs, more dominant M2 activation and lymphangiogenic mediator (VEGF-C) expression (27).

CD200 (a known immunosuppressive surface protein) is overexpressed in stroma around cSCC, mainly by blood vessel endothelia. CD200 is also expressed on cSCC tumor cells (63). In addition, more CD200R^+^ cells are located in the cSCC microenvironment than normal skin, and CD200R was detected on macrophages and dendritic cells (28). Increased CD200 expression on tumor cells is associated with tumor progression and decreased patient survival (63, 64). Endothelial CD200 may inhibit aberrant diapedesis of macrophages during inflammation partly through downregulation of macrophage adhesion molecules. Hence, through this mechanism, CD200 may play a role in suppression of macrophage function (65). Moreover, binding of endothelial CD200 to CD200R on macrophages and dendritic cells inhibits proinflammatory activation (66–70) and suppresses classic activation of macrophages; therefore, M2 cells become the predominant macrophage polarized state (71).

Anti-CD200 antibody (through blocking the CD200-CD200R interaction) has been shown to improve antitumor activity against CD200-expressing human tumors in a mouse model (72, 73). Thus, anti-CD200 therapies could represent effective treatments for aggressive SCCs (28).

## 4 Dendritic cells and Langerhans cells

Dendritic cells (DC) are antigen-presenting cells (APCs) that play an important role in linking the innate and adaptive immune systems (74). The ability of DCs to induce tumor-specific T-cell responses facilitate their vital role in cancer immune surveillance (75).

Three main subsets of cutaneous DCs in humans include Langerhans cells (LCs), myeloid DCs (mDCs), and plasmacytoid DCs (pDCs) (76). As Langerhans cells are found in the epidermis, they are the first APCs to encounter SCC (77). LCs from human SCC can stimulate CD8^+^- or NK-cell-mediated response more efficiently than other DC subsets, resulting in a more robust proliferation of naive CD8^+^ T cells (78).

In addition to the primary role of DCs in initiating the cellular immunity, they are also involved in polarizing the naive CD4^+^ T cells towards a Th2 immune response through releasing type II cytokines, such as IL-4, IL-5, and IL-13 (79). Furthermore, it has been reported that LCs from SCC were more powerful inducers of allogeneic CD4^+^ and CD8^+^ T-cell proliferation and IFN-γ production compared to those from normal skin and eventually more potent in activating type 1 T-cell responses (77).

Tumor-induced dendritic cells dysfunction (29) and tumor-induced DC apoptosis (80–82) are two of major strategies used by tumors to escape immune surveillance.

Several studies have revealed that the number of both LCs and CD11c^+^ dermal DCs is markedly reduced in SCC lesions (83, 84) and the ability of the dermal myeloid DCs to activate T cells and stimulate the production of interferon (IFN)-γ is diminished (83, 85).

Higher levels of immunosuppressive cytokines, such as TGF-*β*, IL-10, IL-6 and VEGF-A, in the microenvironment of SCCs are believed to be possible causes of mDCs suppression (83). IL-10 has the potential to inhibit the differentiation of monocytes to DC (86), weaken APC function of DCs (87, 88), suppress DCs’ ability to activate T cells, and cause induction of antigen-specific anergy (89). Increased VEGF levels are related to decreased number of DCs in tumor lesion and in the peripheral blood of patients with various malignant tumors. This finding demonstrates the ability of VEGF to inhibit DC differentiation (90–92).

**Figure 3:**
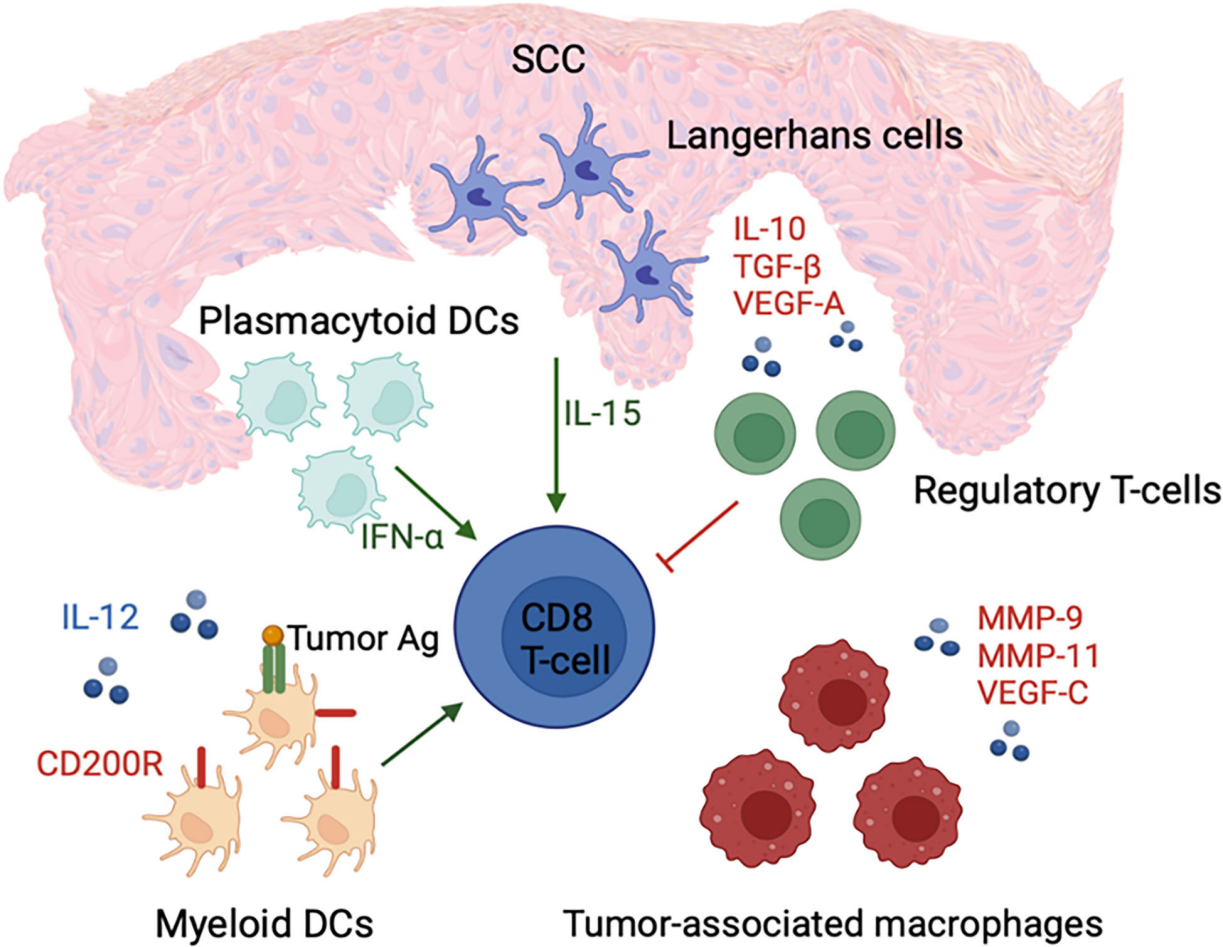
cSCC microenvironment is associated with an increased number of IFN-α-secreting pDCs and LCs with enhanced ability to activate CD8+ T cells, which potentially promote immunosurveillance. In contrast, an increased number of regulatory T cells; tumor-associated macrophages; and immune suppressive cytokines, such as IL-10, TGF-β, and VEGF-A, are present in the tumor microenvironment. These factors contribute to tumor growth and immune dysfunction through suppression of mDC and CD8+ T cell activity.

The presence of large numbers of pDCs is another distinguishing feature of the SCC tumor microenvironment (83). These cells facilitate tumor eradication through production of large quantities of IFN-α in response to foreign antigen. Moreover, pDCs can recognize, process, and cross-present foreign antigen to CD8^+^ T lymphocytes (93, 94). Despite lower antigen uptake by pDCs compared to mDCs, pDCs may still be effective in anti-tumor immune response (**Figure 3**) (95).

It can be concluded that DCs are desirable targets for tumor immunotherapy due to their capacity to link the innate and adaptive immune systems as well as their ability to initiate the immune response (74). In addition, human LCs have been shown to be more potent inducers of type 1 T-cell response in the cSCC microenvironment. Hence, LCs can be used in DC-based cancer immunotherapy as a promising novel strategy in the treatment of skin malignancies (77).

## 5 T-lymphocytes

Numerous immune cells, including T-cells, are found in SCC lesions (96–98). Despite T cell infiltration into cutaneous SCC (cSCC), these cells are incapable of eradicating the tumor (99, 100).

It has been demonstrated that SCC and transplant-associated SCC (TSCC) microenvironments have significantly greater numbers of CD3^+^ and CD8^+^ T cells than normal skin. These cells accumulate predominantly in the peritumoral region and are less frequently noted within the tumoral region. The number of FOXP3^+^ T reg cells is increased in both SCC and TSCC compared to normal skin (101). Approximately more than 50% of the T cells infiltrating cSCCs from both immunocompetent and immunosuppressed patients are FOXP3^+^ T reg cells (97). These cells are CD4^+^ and lack CLA, CCR4, and CCR6 (skin resident T reg markers) (102). Moreover, these cells express markers of central memory T cells, such as L-selectin and CCR7. Given that T reg cells do not proliferate locally in tumors, recruitment from the blood may be the main mechanism responsible for significant presence of these cells in tumors (97).

Although FOXP3^+^ T reg cells contribute to immune tolerance (103), which is important for preventing autoimmune diseases (104), they may suppress antitumor immunity (105, 106) and play a role in immune evasion. Particularly, the immune response can be regulated by T reg cells by suppressing the proliferation and cytokine production of effector T cells (107, 108).

Based on several studies, the greater number of tumor infiltrating T regs is related to poor prognosis and lower survival rates in breast (109), ovarian (110, 111) and gastric carcinomas (105). T regs may contribute to cSCC metastasis and thus have potential prognostic significance (100). Some recent studies have identified CD8^+^ Tregs in cSCC (112) and other tumors (113) that exhibit even stronger regulatory activities compared to CD4^+^ Tregs (114). Given its ability to decrease the number of FOXP3^+^ T reg cells and inhibit T reg cell function, imiquimod could effectively inhibit the immunological destruction of cSCC (97).

**Figure 4:**
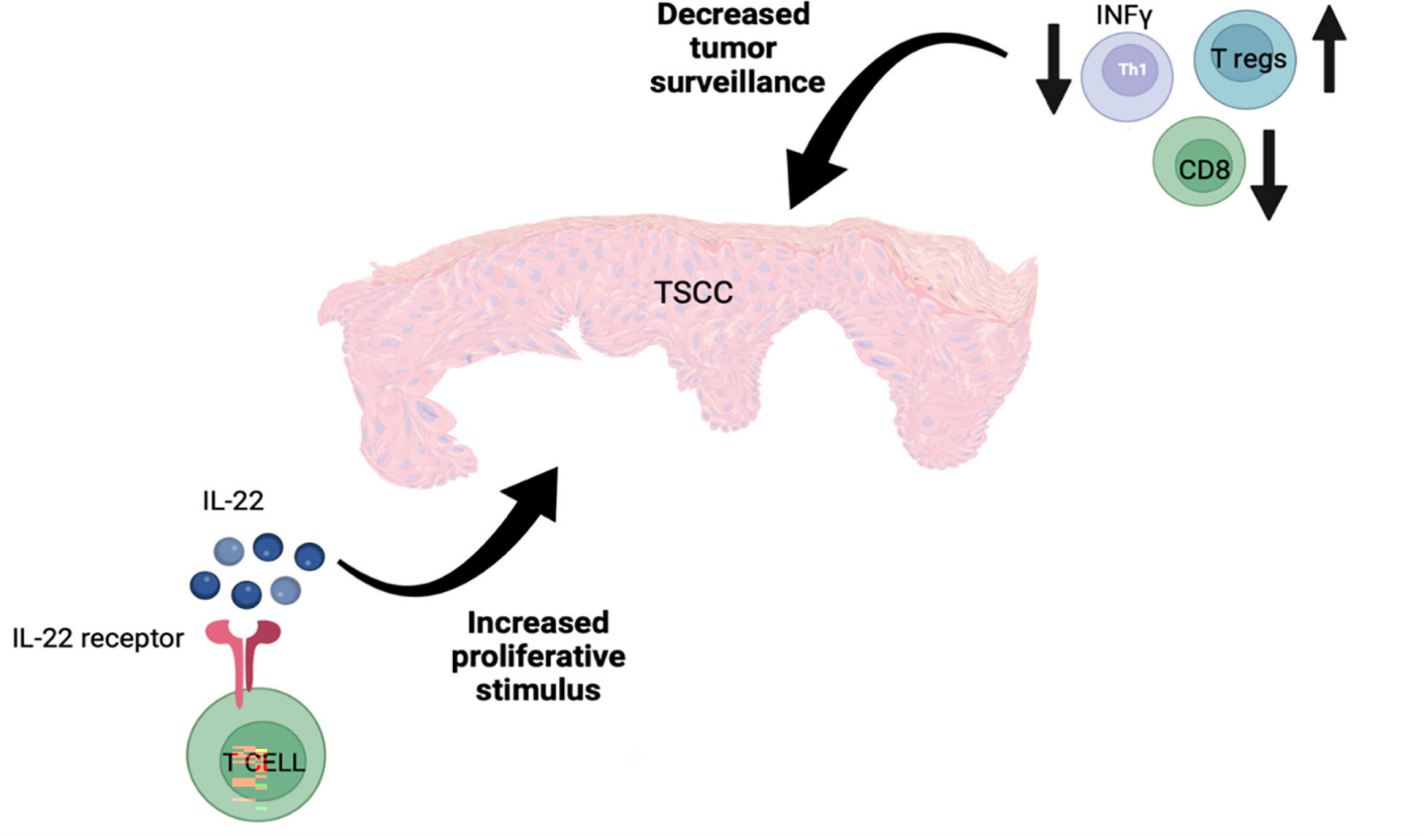
The aggressive nature of TSCC is potentially explained by the presence of increased numbers of T regs along with reduced numbers of CD8+ and IFN-γ-producing T cells, resulting in reduced tumor surveillance as well as an increase in IL-22-producing T cells, which stimulate tumor cell proliferation.

TSCC has a distinct immune microenvironment that promotes tumor growth. There are fewer T cells, especially CD8^+^ T cells, in TSCC lesions in comparison to SCC lesions (101), and a decreased Tc/Treg ratio in TSCC has also been reported (112). Furthermore, an increased number of IL-22 producing CD8^+^ T cells and decreased number of CD4^+^ Th1 T cells have been revealed in TSCC lesions. Higher T regs and lower CD8^+^ T cells, which result in decreased immune surveillance, and increased exposure to IL-22, which enhances tumor proliferation, represent two main factors that contribute to the aggressive nature of TSCC (101) (**Figure 4**).

Compared to photodamaged skin, SCCs are associated with an increased number of CD4^+^ T-cells. However, compared to premalignant lesions, including intraepidermal carcinoma (IEC), SCCs may also be associated with fewer numbers of CD8^+^ T-cells. The ratio of CD4^+^ to CD8^+^ T-cells is significantly increased in SCC compared to IEC (115).

## 6 Lymphatic and blood vessels

The lymphatic vascular system is the main pathway for metastatic spread in SCCs. Various cancers can cause lymphangiogenesis, which is associated with increased expression of vascular endothelial growth factors as well as increased relative lymphatic vessel area (LVA) or lymphatic vessel density (LVD) (59, 116, 117) In this context, overexpression of genes related to lymphangiogenesis and increased LVD has been shown in cSCC compared to normal skin (118).

The risk of metastasis in SCCs is related to several variables, including tumor thickness, horizontal tumor size, and desmoplastic growth (11, 15–17). Tumor thickness has been shown to be the most accurate predictive factor for metastasis in SCCs. Metastatic SCCs are associated with increased lymphangiogenesis; however, the extent depends on the thickness of the tumor. It has been shown that greater tumor thickness in SCCs is accompanied by an increase in relative lymphatic vessel area and lymphatic vessel density (118). Despite clear excision margins in SCCs, increased dermal lymphangiogenesis can facilitate metastatic spread (59).

VEGF-C is a key lymphangiogenesis mediator (119). Increased VEGF-C levels in the tumor and the juxtatumoral dermis of cSCC compared with normal skin have been reported, and it has been suggested that tumor-associated macrophages may play an important role in lymphangiogenesis through production of VEGF-C (59).

Podoplanin is a distinctive immunohistochemical marker of lymphatic endothelial cells. Overexpression of podoplanin in both tumor cells and stroma of cSCC have been reported (120). Additionally, a positive correlation is noted between the expression of podoplanin in intratumoral and peritumoral regions of cSCC and the Broder’s tumor differentiation grades (121–123) as well as the depth of tumor invasion to the dermis based on the Clark’s scale (124). According to several studies, increased podoplanin expression is associated with a higher mean of LVD in the SCC microenvironment (120, 124–126) and presence of LN metastasis in SCC patients (120, 121, 127, 128). Therefore, podoplanin could be used as a predictor of SCC prognosis given that increased podoplanin expression is related to poor prognosis and decreased survival in cSCC patients (120).

Most immune cells have their first contact with a tumor through endothelial cells of the local blood vessels (28). Endothelial cell integrity is believed to play an important role in tumors. Normal endothelial cells promote homeostasis, but dysfunctional endothelial cells can lead to cancer growth (129). Abnormal angiogenesis also contributes to tumor growth and promotes metastatic spread. The density of neovascularization in cSCC is positively correlated with deeper invasions and poorer tumor differentiation. As a result, SCC tumors with high angiogenic activity are classified as aggressive with poor prognosis (130). Podoplanin represents a potential target for antimetastatic therapy in cSCC. A cancer-specific monoclonal antibody against human podoplanin has been demonstrated to be an effective treatment strategy particularly in podoplanin-expressing malignancies (131).

## 7 Cytokines

Cytokines play an important role in tumor biology. It was previously thought that IFN-γ and other Th1 cytokines exhibit antitumor activity, whereas IL-4 and other Th2 cytokines have protumor function (132). However, based on recent studies, some cytokines, such as IFN-γ, have been shown to have pro-tumor or anti-tumor functions depending on the tumor type and tumor microenvironment (133).

High serum levels of proinflammatory cytokines, such as interleukin (IL)-1, IL-6, IL-8, and TNF-α, are often related to tumor growth and poor clinical prognosis in cancer patients (134–137). It has been suggested that the balance between multiple cytokines may contribute to the SCC pathogenesis (138). Several cytokines, including IL-6, IFN-γ, TGF-β and GM-CSF, play a role in keratinocyte proliferation and SCC development (139–143).

Significantly elevated serum IFN-γ levels have been reported in SCC patients compared with normal subjects, and higher IFN-γ levels in SCC patients are corelated with more advanced cancer stages. The combination of serum IFN-γ and TGF-β levels is more reliable for diagnosis of SCC, whereas measurement of serum IFN-γ alone is helpful in evaluating the SCC progression from early to middle stages (138).

Elevated serum IL-6 levels are associated with increased malignancy and poor prognosis in different types of tumors (144–146). It has been demonstrated that IL-6 is important in transforming benign tumors into malignant, invasive SCCs in the HaCaT cell model of skin carcinogenesis. A complex, reciprocally regulated cytokine network induced by IL-6 in the tumor cells, including inflammatory cytokines (MCP-1, GM-CSF, and IL-8) and angiogenic factor (VEGF), results in malignant and invasive tumor growth *in vivo* and stimulates tumor cell proliferation and migrations. These findings indicate that IL-6 could represent a great target for effective cSCC treatment (147).

IL-24 overexpression has been noted in invasive cSCC. IL-24 facilitates cSCC invasion (132) by increasing focal MMP-7 expression, and MMP-7 promotes cancer cell proliferation, migration, and invasion (148).

According to several reports, constitutive expression of G-CSF and GM-CSF together has been shown in SCCs (149–151). Through induction of cell proliferation, migration, and angiogenesis in cSCCs, G-CSF and GM-CSF contribute to tumor growth, invasion, and metastasis (149, 150, 152).

Transforming growth factor-β (TGF-β) signaling is mediated by several downstream proteins, such as Smad family proteins. This signaling pathway has a paradoxical role by acting as a tumor-suppressor or tumor-promoting factor in many types of cancers, such as SCC. In the early stages of SCC, TGF-β1 and TGF-βRI act as tumor suppressors. However, in later stages, these proteins promote tumor growth. Smad2, TGF-βRII, and Smad4 are typically considered tumor suppressors in SCC (153).

IL-22 is produced by CD4^+^ helper T lymphocytes (Th), such as Th1, Th17, and Th22 as well as a subset of CD8^+^ cytotoxic T cells (Tc22) (154–157). Significantly increased IL-22 is noted in the peritumoral regions of SCC and TSCC compared to normal skin. In transplant patients, overexpression of IL-22 and IL-22R facilitate tumor growth (101) and result in poorer prognosis (158). In addition to the role of IL-22 in cell proliferation, it can reduce IFN-γ production by Th1 cells as well as increase the production of immunosuppressive cytokines (159). It has been proposed that treating highly aggressive forms of SCCs in transplant patients by targeting the IL-22 pathway could represent an important, life-saving strategy (101).

## 8 Discussion

Skin malignancies are the most prevalent human cancers, and the immune system plays an important role in their development, progression, and eradication (160). There are approximately 1 million memory T cells/cm^2^ in normal human skin, which is approximately twofold the number of T cells that exist in the entire circulation (161), indicating the importance of cutaneous immune surveillance as part of the immune system.

The immune microenvironment surrounding the cSCC is dynamic and contains contradictory forces that promote and suppress tumor growth (72, 162–165).

To summarize, the cSCC microenvironment has more Tregs and myeloid-derived suppressor cells that suppress immune responses and fewer mDCs with poor antigen-presenting function. The macrophages present in the cSCC microenvironment predominantly exhibit the M2 phenotype and promote tumor invasion and metastasis through producing MMPs and lymphangiogenic mediators. The SCC microenvironment is rich in IL-6, IFN-γ, TGF-β, GM-CSF, and IL-24, which induce tumor growth and invasion. Moreover, increased dermal lymphangiogenesis facilitates metastatic spread. Overexpression of IL-22 and IL-22R accelerate tumor proliferation and subsequently result in poorer prognosis in transplant patients with cSCCs.

## Author contributions

VS performed literature searches and composed initial draft of the manuscript. ND co-wrote initial draft and participated in all revisions. JAC conceived the original concept and provided multiple revisions of the manuscript. All authors contributed to the article and approved the submitted version.

## References

[1] The skin cancer foundation, in: Our new approach to a challenging skin cancer statistic (2021). Available at: https://www.skincancer.org/blog/our-new-approach-to-a-challenging-skin-cancer-statistic/ (Accessed 18 October,2022).

[2] Lucas R McMichael T Smith W Armstrong B. Solar ultraviolet radiation: Global burden of disease from solar ultraviolet radiation. Environ Burden Dis Ser (2006) 13).

[3] Gray D Suman V Su W Clay R Harmsen W Roenigk R. Trends in the population-based incidence of squamous cell carcinoma of the skin first diagnosed between 1984 and 1992. Arch Dermatol (1997) 133(6):735–40. 9197827

[4] Di Nardo L Pellegrini C Di Stefani A Del Regno L Sollena P Piccerillo A. Molecular genetics of cutaneous squamous cell carcinoma: Perspective for treatment strategies. J Eur Acad Dermatol Venereol (2020) 34(5):932–41. doi: 10.1111/jdv.16098 31747091

[5] Chang D Shain AH. The landscape of driver mutations in cutaneous squamous cell carcinoma. NPJ Genom Med (2021) 6(1):61. doi: 10.1038/s41525-021-00226-4 34272401 PMC8285521

[6] Durinck S Ho C Wang NJ Liao W Jakkula LR Collisson EA. Temporal dissection of tumorigenesis in primary cancers. Cancer Discov (2011) 1(2):137–43. doi: 10.1158/2159-8290.CD-11-0028 PMC3187561 21984974

[7] Li YY Hanna GJ Laga AC Haddad RI Lorch JH Hammerman PS. Genomic analysis of metastatic cutaneous squamous cell carcinoma. Clin Cancer Res (2015) 21(6):1447–56. doi: 10.1158/1078-0432.CCR-14-1773 PMC4359951 25589618

[8] Pickering CR Zhou JH Lee JJ Drummond JA Peng SA Saade RE. Mutational landscape of aggressive cutaneous squamous cell carcinoma. Clin Cancer Res (2014) 20(24):6582–92. doi: 10.1158/1078-0432.CCR-14-1768 PMC4367811 25303977

[9] Fu T Aasi SZ Hollmig ST. Management of high-risk squamous cell carcinoma of the skin. Curr Treat Options Oncol (2016) 17(7):34. doi: 10.1007/s11864-016-0408-2 27262708

[10] Weinberg A Ogle C Shim E. Metastatic cutaneous squamous cell carcinoma: An update. Dermatol Surg (2007) 33(8):885–99. doi: 10.1111/j.1524-4725.2007.33190.x 17661931

[11] Brantsch KD Meisner C Schonfisch B Trilling B Wehner-Caroli J Rocken M. Analysis of risk factors determining prognosis of cutaneous squamous-cell carcinoma: A prospective study. Lancet Oncol (2008) 9(8):713–20. doi: 10.1016/S1470-2045(08)70178-5 18617440

[12] Lansbury L Bath-Hextall F Perkins W Stanton W Leonardi-Bee J. Interventions for non-metastatic squamous cell carcinoma of the skin: Systematic review and pooled analysis of observational studies. BMJ (2013) 347:f6153. doi: 10.1136/bmj.f6153 24191270 PMC3816607

[13] Hollestein LM de Vries E Nijsten T. Trends of cutaneous squamous cell carcinoma in the Netherlands: Increased incidence rates, but stable relative survival and mortality 1989-2008. Eur J Cancer (2012) 48(13):2046–53. doi: 10.1016/j.ejca.2012.01.003 22342554

[14] Karia PS Jambusaria-Pahlajani A Harrington DP Murphy GF Qureshi AA Schmults CD. Evaluation of American joint committee on cancer, international union against cancer, and Brigham and women's hospital tumor staging for cutaneous squamous cell carcinoma. J Clin Oncol (2014) 32(4):327–34. doi: 10.1200/JCO.2012.48.5326 PMC3897257 24366933

[15] Kwon S Dong ZM Wu PC. Sentinel lymph node biopsy for high-risk cutaneous squamous cell carcinoma: Clinical experience and review of literature. World J Surg Oncol (2011) 9:80. doi: 10.1186/1477-7819-9-80 21771334 PMC3156743

[16] Reschly MJ Messina JL Zaulyanov LL Cruse W Fenske NA. Utility of sentinel lymphadenectomy in the management of patients with highrisk cutaneous squamous cell carcinoma. Dermatologic Surg (2003) 29:135–40. doi: 10.1046/j.1524-4725.2003.29035.x 12562341

[17] Rowe DE Carroll RJ Day C. Prognostic factors for local recurrence, metastasis, and survival rates in squamous cell carcinoma of the skin, ear, and lip. Implications Treat Modality Selection. J Am Acad Dermatol (1992) 26(6):976–90. doi: 10.1016/0190-9622(92)70144-5 1607418

[18] Veness M Morgan G Palme C Gebski V. Surgery, and adjuvant radiotherapy in patients with cutaneous head and neck squamous cell carcinoma metastatic to lymph nodes: Combined treatment should be considered best practice. Laryngoscope (2005) 115(5):870–5. doi: 10.1097/01.MLG.0000158349.64337.ED 15867656

[19] Keeping S Xu Y Chen CI Cope S Mojebi A Kuznik A. Comparative efficacy of cemiplimab versus other systemic treatments for advanced cutaneous squamous cell carcinoma. Future Oncol (2021) 17(5):611–27. doi: 10.2217/fon-2020-0823 33052055

[20] Villani A Ocampo-Garza SS Potestio L Fabbrocini G Ocampo-Candiani J Ocampo-Garza J. Cemiplimab for the treatment of advanced cutaneous squamous cell carcinoma. Expert Opin Drug Saf (2022) 21(1):21–9. doi: 10.1080/14740338.2022.1993819 34644510

[21] Keytruda® (Pembrolizumab) injection, for intravenous use. NJ: Whitehouse Station: Merck & Co. I (2021).

[22] Oro-Ayude M Suh-Oh HJ Sacristan-Santos V Vazquez-Bartolome P Florez A. Nivolumab for metastatic cutaneous squamous cell carcinoma. Case Rep Dermatol (2020) 12(1):37–41. doi: 10.1159/000505478 32595466 PMC7315375

[23] Fujimura T Kambayashi Y Tono H Lyu C Ohuchi K Hashimoto A. Successful treatment of unresectable recurrent cutaneous squamous cell carcinoma of the scalp with meningeal invasion with nivolumab monotherapy. Dermatol Ther (2020) 33(4):e13672. doi: 10.1111/dth.13672 32449226

[24] Maruhashi T Sugiura D Okazaki IM Okazaki T. Lag-3: From molecular functions to clinical applications. J Immunother Cancer (2020) 8(2). doi: 10.1136/jitc-2020-001014 PMC7488795 32929051

[25] Wang M Du Q Jin J Wei Y Lu Y Li Q. Lag3 and its emerging role in cancer immunotherapy. Clin Transl Med (2021) 11(3):e365. doi: 10.1002/ctm2.365 33784013 PMC7989707

[26] Alberti A Bossi P. Immunotherapy for cutaneous squamous cell carcinoma: Results and perspectives. Front Oncol (2021) 11:727027. doi: 10.3389/fonc.2021.727027 35070956 PMC8766667

[27] Ovits CG Carucci JA. Immune environment of cutaneous malignancies. In: AAGe al, editor. Clinical and basic immunodermatology. Switzerland: Springer International Publishing (2017). p. 741–55.

[28] Belkin DA Mitsui H Wang CQ Gonzalez J Zhang S Shah KR. Cd200 upregulation in vascular endothelium surrounding cutaneous squamous cell carcinoma. JAMA Dermatol (2013) 149(2):178–86. doi: 10.1001/jamadermatol.2013.1609 23560298

[29] Pinzon-Charry A Maxwell T Lopez JA. Dendritic cell dysfunction in cancer: A mechanism for immunosuppression. Immunol Cell Biol (2005) 83(5):451–61. doi: 10.1111/j.1440-1711.2005.01371.x 16174093

[30] Mittal D Gubin MM Schreiber RD Smyth MJ. New insights into cancer immunoediting and its three component phases elimination, equilibrium and escape. Curr Opin Immunol (2014) 27:16–25. doi: 10.1016/j.coi.2014.01.004 24531241 PMC4388310

[31] Seliger B. Novel insights into the molecular mechanisms of hla class I abnormalities. Cancer Immunol Immunother (2012) 61(2):249–54. doi: 10.1007/s00262-011-1153-9 PMC11029063 22120755

[32] Whiteside TL. Tumor-induced death of immune cells: Its mechanisms and consequences. Semin Cancer Biol (2002) 12(1):43–50. doi: 10.1006/scbi.2001.0402 11926411

[33] van Kempen LC Ruiter DJ van Muijen GN Coussens LM. The tumor microenvironment: A critical determinant of neoplastic evolution. Eur J Cell Biol (2003) 82(11):539–48. doi: 10.1078/0171-9335-00346 14703010

[34] Elmusrati A Wang J Wang CY. Tumor microenvironment and immune evasion in head and neck squamous cell carcinoma. Int J Oral Sci (2021) 13(1):24. doi: 10.1038/s41368-021-00131-7 34341329 PMC8329257

[35] Bronte V Brandau S Chen SH Colombo MP Frey AB Greten TF. Recommendations for myeloid-derived suppressor cell nomenclature and characterization standards. Nat Commun (2016) 7:12150. doi: 10.1038/ncomms12150 27381735 PMC4935811

[36] Veglia F Sanseviero E Gabrilovich DI. Myeloid-derived suppressor cells in the era of increasing myeloid cell diversity. Nat Rev Immunol (2021) 21(8):485–98. doi: 10.1038/s41577-020-00490-y PMC7849958 33526920

[37] Wang PF Song SY Wang TJ Ji WJ Li SW Liu N. Prognostic role of pretreatment circulating mdscs in patients with solid malignancies: A meta-analysis of 40 studies. Oncoimmunology (2018) 7(10):e1494113. doi: 10.1080/2162402X.2018.1494113 30288362 PMC6169582

[38] Condamine T Mastio J Gabrilovich DI. Transcriptional regulation of myeloid-derived suppressor cells. J Leukoc Biol (2015) 98(6):913–22. doi: 10.1189/jlb.4RI0515-204R PMC4661041 26337512

[39] Nagaraj S Gabrilovich DI. Myeloid-derived suppressor cells in human cancer. Cancer J (2010) 16(4):348–53. doi: 10.1097/PPO.0b013e3181eb3358 20693846

[40] Gabrilovich D Nagaraj S. Myeloid-derived suppressor cells as regulators of the immune system. Nat Rev Immunol (2009) 9:162–74. doi: 10.1038/nri2506 PMC2828349 19197294

[41] Gehad AE Lichtman MK Schmults CD Teague JE Calarese AW Jiang Y. Nitric oxide-producing myeloid-derived suppressor cells inhibit vascular e-selectin expression in human squamous cell carcinomas. J Invest Dermatol (2012) 132(11):2642–51. doi: 10.1038/jid.2012.190 PMC3449043 22718118

[42] Nefedova Y Fishman M Sherman S Wang X Beg AA Gabrilovich DI. Mechanism of all-trans retinoic acid effect on tumor-associated myeloid-derived suppressor cells. Cancer Res (2007) 67:11021–8. doi: 10.1158/0008-5472.CAN-07-2593 18006848

[43] Kusmartsev S Cheng F Yu B Nefedova Y Sotomayor E Lush R. All-Trans-Retinoic acid eliminates immature myeloid cells from tumor-bearing mice and improves the effect of vaccination. Cancer Res (2003) 63:4441–9. 12907617

[44] Iclozan C Antonia S Chiappori A Chen DT Gabrilovich D. Therapeutic regulation of myeloid-derived suppressor cells and immune response to cancer vaccine in patients with extensive stage small cell lung cancer. Cancer Immunol Immunother (2013) 62(5):909–18. doi: 10.1007/s00262-013-1396-8 PMC3662237 23589106

[45] Tobin RP Jordan KR Robinson WA Davis D Borges VF Gonzalez R. Targeting myeloid-derived suppressor cells using all-trans retinoic acid in melanoma patients treated with ipilimumab. Int Immunopharmacol (2018) 63:282–91. doi: 10.1016/j.intimp.2018.08.007 PMC6134177 30121453

[46] Wang YC He F Feng F Liu XW Dong GY Qin HY. Notch signaling determines the M1 versus M2 polarization of macrophages in antitumor immune responses. Cancer Res (2010) 70(12):4840–9. doi: 10.1158/0008-5472.CAN-10-0269 20501839

[47] Qian BZ Pollard JW. Macrophage diversity enhances tumor progression and metastasis. Cell (2010) 141(1):39–51. doi: 10.1016/j.cell.2010.03.014 20371344 PMC4994190

[48] Noy R Pollard JW. Tumor-associated macrophages: From mechanisms to therapy. Immunity (2014) 41(1):49–61. doi: 10.1016/j.immuni.2014.06.010 25035953 PMC4137410

[49] Pettersen JS Fuentes-Duculan J Suarez-Farinas M Pierson KC Pitts-Kiefer A Fan L. Tumor-associated macrophages in the cutaneous scc microenvironment are heterogeneously activated. J Invest Dermatol (2011) 131(6):1322–30. doi: 10.103/jid.2011.9 PMC3334331 21307877

[50] Mills CD Kincaid K Alt JM Heilman MJ Hill AM. M-1/M-2 macrophages and the Th1/Th2 paradigm. J Immunol (2000) 164(12):6166–73. doi: 10.4049/jimmunol.164.12.6166 10843666

[51] Trinchieri G. Interleukin-12 and the regulation of innate resistance and adaptive immunity. Nat Rev Immunol (2003) 3(2):133–46. doi: 10.1038/nri1001 12563297

[52] Edwards JP Zhang X Frauwirth KA Mosser DM. Biochemical and functional characterization of three activated macrophage populations. J Leukoc Biol (2006) 80(6):1298–307. doi: 10.1189/jlb.0406249 PMC2642590 16905575

[53] Gocheva V Wang HW Gadea BB Shree T Hunter KE Garfall AL. Il-4 induces cathepsin protease activity in tumor-associated macrophages to promote cancer growth and invasion. Genes Dev (2010) 24(3):241–55. doi: 10.1101/gad.1874010 PMC2811826 20080943

[54] Sica A Mantovani A. Macrophage plasticity and polarization: *In vivo* veritas. J Clin Invest (2012) 122(3):787–95. doi: 10.1172/JCI59643 PMC3287223 22378047

[55] Pinto CA Carvalho PE Antonangelo L Garippo A Da Silva AG Soares F. Morphometric evaluation of tumor matrix metalloproteinase 9 predicts survival after surgical resection of adenocarcinoma of the lung. Clin Cancer Res (2003) 9(8):3098–104. 12912961

[56] Buergy D Weber T Maurer GD Mudduluru G Medved F Leupold JH. Urokinase receptor, mmp-1 and mmp-9 are markers to differentiate prognosis, adenoma and carcinoma in thyroid malignancies. Int J Cancer (2009) 125(4):894–901. doi: 10.1002/ijc.24462 19480010

[57] Shah SA Spinale FG Ikonomidis JS Stroud RE Chang EI Reed CE. Differential matrix metalloproteinase levels in adenocarcinoma and squamous cell carcinoma of the lung. J Thorac Cardiovasc Surg (2010) 139(4):984–90. doi: 10.1016/j.jtcvs.2009.12.016 PMC2844342 20304142

[58] Zhao ZS Chu YQ Ye ZY Wang YY Tao HQ. Overexpression of matrix metalloproteinase 11 in human gastric carcinoma and its clinicopathologic significance. Hum Pathol (2010) 41(5):686–96. doi: 10.1016/j.humpath.2009.10.010 20060156

[59] Moussai D Mitsui H Pettersen JS Pierson KC Shah KR Suárez-Fariñas M. The human cutaneous squamous cell carcinoma microenvironment is characterized by increased lymphatic density and enhanced expression of macrophage-derived vegf-c. J Invest Dermatol (2011) 131(1):229–36. doi: 10.1038/jid.2010.266 20827282

[60] Boone B Blokx W De Bacquer D Lambert J Ruiter D Brochez L. The role of vegf-c staining in predicting regional metastasis in melanoma. Virchows Arch (2008) 453(3):257–65. doi: 10.1007/s00428-008-0641-6 18679715

[61] Sugiura T Inoue Y Matsuki R Ishii K Takahashi M Abe M. Vegf-c and vegf-d expression is correlated with lymphatic vessel density and lymph node metastasis in oral squamous cell carcinoma: Implications for use as a prognostic marker. Int J Oncol (2009) 34(3):673–80. doi: 10.3892/ijo_00000193 19212672

[62] Jiang X Wang M Cyrus N Yanez DA Lacher RK Rhebergen AM. Human keratinocyte carcinomas have distinct differences in their tumor-associated macrophages. Heliyon (2019) 5(8). doi: 10.1016/j.heliyon.2019.e02273 PMC6706589 31463392

[63] Stumpfova M Ratner D Desciak EB Eliezri YD Owens DM. The immunosuppressive surface ligand Cd200 augments the metastatic capacity of squamous cell carcinoma. Cancer Res (2010) 70(7):2962–72. doi: 10.1158/0008-5472 PMC2848906 20332223

[64] Khan IZ Del Guzzo CA Shao A Cho J Du R Cohen AO. The Cd200-Cd200r axis promotes squamous cell carcinoma metastasis *Via* regulation of cathepsin K. Cancer Res (2021) 81(19):5021–32. doi: 10.1158/0008-5472.CAN-20-3251 PMC8488015 34183355

[65] Ko YC Chien HF Jiang-Shieh YF Chang CY Pai MH Huang JP. Endothelial Cd200 is heterogeneouslydistributed, regulated and involved in immune cell-endothelium interactions. J Anat (2009) 214(1):183–95. doi: 10.1111/j.1469-7580.2008.00986.x PMC2667927 19166481

[66] Hoek RM Ruuls SR Murphy CA Wright GJ Goddard R Zurawski SM. Down-regulation of the macrophage lineage through interaction with Ox2 (Cd200). Science (2000) 290(5497):1768– 71. doi: 10.1126/science.290.5497.1768 11099416

[67] Gorczynski RM Cattral MS Chen Z Hu J Lei J Min WP. An immunoadhesin incorporating the molecule ox-2 is a potent immunosuppressant that prolongs allo- and xenograft survival. J Immunol (1999) 163(3):1654–60. 10415071

[68] Taylor N McConachie K Calder C Dawson R Dick A Sedgwick JD. Enhanced tolerance to autoimmune uveitis in Cd200-deficient mice correlates with a pronounced Th2 switch in response to antigen challenge. J Immunol (2005) 174(1):143–54. doi: 10.4049/jimmunol.174.1.143 PMC2446433 15611236

[69] Gorczynski RM Yu K Clark D. Receptor engagement on cells expressing a ligand for the tolerance-inducing molecule Ox2 induces an immunoregulatory population that inhibits alloreactivity in vitro and in vivo. J Immunol (2000) 165(9):4854–60. doi: 10.4049/jimmunol.165.9.4854 11046009

[70] Koning N van Eijk M Pouwels W Brouwer MS Voehringer D Huitinga I. Expression of the inhibitory Cd200 receptor is associated with alternative macrophage activation. J Innate Immun (2010) 2(2):195–200. doi: 10.1159/000252803 20375636

[71] Zhang S Cherwinski H Sedgwick JD Phillips JH. Molecular mechanisms of Cd200 inhibition of mast cell activation. J Immunol (2004) 173(11):6786–93. doi: 10.4049/jimmunol.173.11.6786 15557172

[72] Kretz-Rommel A Qin F Dakappagari N Ravey EP McWhirter J Oltean D. Cd200 expression on tumor cells suppresses antitumor immunity: New approaches to cancer immunotherapy. J Immunol (2007) 178(9):5595–605. doi: 10.4049/jimmunol.178.9.5595 17442942

[73] Kretz-Rommel A Qin F Dakappagari N Cofiell R Faas SJ Bowdish KS. Blockade of Cd200 in the presence or absence of antibody effector function: Implications for anti-Cd200 therapy. J Immunol (2008) 180(2):699–705. doi: 10.4049/jimmunol.180.2.699 18178807

[74] Yanofsky VR Mitsui H Felsen D Carucci JA. Understanding dendritic cells and their role in cutaneous carcinoma and cancer immunotherapy. J Immunol Res (2013) 2013:624123. doi: 10.1155/2013/624123 PMC3625554 23606870

[75] Gottfried E Kreutz M Mackensen A. Tumor-induced modulation of dendritic cell function. Cytokine Growth Factor Rev (2008) 19(1):65–77. doi: 10.1016/j.cytogfr.2007.10.008 18061513

[76] Zaba LC Krueger JG Lowes MA. Resident and "Inflammatory" dendritic cells in human skin. J Invest Dermatol (2009) 129(2):302–8. doi: 10.1038/jid.2008.225 PMC2746703 18685620

[77] Fujita H Suarez-Farinas M Mitsui H Gonzalez J Bluth MJ Zhang S. Langerhans cells from human cutaneous squamous cell carcinoma induce strong type 1 immunity. J Invest Dermatol (2012) 132(6):1645–55. doi: 10.1038/jid.2012.34 PMC3677713 22402444

[78] Klechevsky E Morita R Liu M Cao Y Coquery S Thompson-Snipes L. Functional specializations of human epidermal langerhans cells and Cd14+ dermal dendritic cells. Immunity (2008) 29(3):497–510. doi: 10.1016/j.immuni.2008.07.013 18789730 PMC2688399

[79] Banchereau J Klechevsky E Schmitt N Morita R Palucka K Ueno H. Harnessing human dendritic cell subsets to design novel vaccines. Ann N Y Acad Sci (2009) 1174:24–32. doi: 10.1111/j.1749-6632.2009.04999.x 19769733 PMC2759267

[80] Esche C Lokshin A Shurin GV Gastman BR Rabinowich H Watkins SC. Tumor's other immune targets: Dendritic cells. J Leukoc Biol (1999) 66(2):336–44. doi: 10.1002/jlb.66.2.336 10449178

[81] Pirtskhalaishvili G Shurin GV Esche C Cai Q Salup RR Bykovskaia SN. Cytokine-mediated protection of human dendritic cells from prostate cancer-induced apoptosis is regulated by the bcl-2 family of proteins. Br J Cancer (2000) 83:506–13. doi: 10.1054/bjoc.2000.1289 PMC2374651 10945499

[82] Pirtskhalaishvili G Shurin GV Gambotto A Esche C Wahl M Yurkovetsky ZR. Transduction of dendritic cells with bcl-xl increases their resistance to prostate cancer-induced apoptosis and antitumor effect in mice. J Immunol (2000) 165(4):1956–64. doi: 10.4049/jimmunol.165.4.1956 10925278

[83] Bluth MJ Zaba LC Moussai D Suarez-Farinas M Kaporis H Fan L. Myeloid dendritic cells from human cutaneous squamous cell carcinoma are poor stimulators of T-cell proliferation. J Invest Dermatol (2009) 129(10):2451–62. doi: 10.1038/jid.2009.96 PMC2846605 19387481

[84] Galan A Ko CJ. Langerhans cells in squamous cell carcinoma vs. pseudoepitheliomatous hyperplasia of the skin. J Cutan Pathol (2007) 34(12):950–2. doi: 10.1111/j.1600-0560.2007.00741.x 18001421

[85] Nestle FO Burg G Fäh J Wrone-Smith T Nickoloff BJ. Human sunlight-induced basal-cell carcinoma-associated dendritic cells are deficient in T cell Co-stimulatory molecules and are impaired as antigen-presenting cells. Am J Pathol (1997) 150(2):641–51. PMC1858265 9033277

[86] Allavena P Piemonti L Longoni D Bernasconi S Stoppacciaro A Ruco L. Il-10 prevents the differentiation of monocytes to dendritic cells but promotes their maturation to macrophages. Eur J Immunol (1998) 28:359–69. doi: 10.1002/(SICI)1521-4141(199801)28:01<359::AID-IMMU359>3.0.CO;2-4 9485215

[87] Buelens C Verhasselt V De Groote D Thielemans K Goldman M Willems F. Interleukin-10 prevents the generation of dendritic cells from human peripheral blood mononuclear cells cultured with interleukin-4 and Granulocyte/Macrophage-colonystimulating factor. Eur J Immunol (1997) 27:756–62. doi: 10.1002/eji.1830270326 9079819

[88] Enk AH Angeloni VL Udey MC Katz SI. Inhibition of langerhans cell antigen-presenting function by il-10. A Role Il-10 Induction Tolerance. J Immunol (1993) 151(5):2390–8. 8103065

[89] Steinbrink K Jonuleit H Muller G Schuler G Knop J Enk A. Interleukin-10 treated human dendritic cells induce a melanomaantigen-specific anergy in Cd8(+) T cells resulting in a failure to lyse tumor cells. Blood (1999) 93:1634–42. 10029592

[90] Lissoni P Malugani F Bonfanti A Bucovec R Secondino S Brivio F. Abnormally enhanced blood concentrations of vascular endothelial growth factor (Vegf) in metastatic cancer patients and their relation to circulating dendritic cells, il-12 and endothelin-1. J Biol Regul Homeost (2001) 15:140–44. 11501971

[91] Takahashi A Kono K Ichihara F Sugai H Fujii H Matsumoto Y. Vascular endothelial growth factor inhibits maturation of dendritic cells induced by lipopolysaccharide, but not by proinflammatory cytokines. Cancer Immunol Immunother (2004) 53(6):543–50. doi: 10.1007/s00262-003-0466-8 PMC11034272 14666382

[92] Saito H Tsujitani S Ikeguchi M Maeta M Kaibara N. Relationship between the expression of vascular endothelial growth factor and the density of dendritic cells in gastric adenocarcinoma tissue. Br J Cancer (1998) 78(12):1573–7. doi: 10.1038/bjc.1998.725 PMC2063230 9862566

[93] Hoeffel G Ripoche AC Matheoud D Nascimbeni M Escriou N Lebon P. Antigen crosspresentation by human plasmacytoid dendritic cells. Immunity (2007) 27(3):481–92. doi: 10.1016/j.immuni.2007.07.021 17869134

[94] Tel J Schreibelt G Sittig SP Mathan TS Buschow SI Cruz LJ. Human plasmacytoid dendritic cells efficiently cross-present exogenous ags to Cd8+ T cells despite lower Ag uptake than myeloid dendritic cell subsets. Blood (2013) 121(3):459–67. doi: 10.1182/blood-2012-06-435644 23212525

[95] Tel J Aarntzen EH Baba T Schreibelt G Schulte BM Benitez-Ribas D. Natural human plasmacytoid dendritic cells induce antigen-specific T-cell responses in melanoma patients. Cancer Res (2013) 73(3):1063–75. doi: 10.1158/0008-5472.CAN-12-2583 23345163

[96] Hussein MR Ahmed RA. Analysis of the mononuclear inflammatory cell infiltrate in the non-tumorigenic, pre-tumorigenic and tumorigenic keratinocytic hyperproliferative lesions of the skin. Cancer Biol Ther (2005) 4(8):819–21. doi: 10.4161/cbt.4.8.1864 16210913

[97] Clark RA Huang SJ Murphy GF Mollet IG Hijnen D Muthukuru M. Human squamous cell carcinomas evade the immune response by down-regulation of vascular e-selectin and recruitment of regulatory T cells. J Exp Med (2008) 205(10):2221–34. doi: 10.1084/jem.20071190 PMC2556796 18794336

[98] Kosmidis M Dziunycz P Suarez-Farinas M Muhleisen B Scharer L Lauchli S. Immunosuppression affects Cd4+ mrna expression and induces Th2 dominance in the microenvironment of cutaneous squamous cell carcinoma in organ transplant recipients. J Immunother (2010) 33(5):538–46. doi: 10.1097/CJI.0b013e3181cc2615 20463594

[99] Halliday GM Patel A Hunt MJ Tefany FJ Barnetson RS. Spontaneous regression of human Melanoma/Nonmelanoma skin cancer: Association with infiltrating Cd4+ T cells. World J Surg (1995) 19:352 – 8. doi: 10.1007/BF00299157 7638987

[100] Lai C August S Albibas A Behar R Cho SY Polak ME. Ox40+ regulatory T cells in cutaneous squamous cell carcinoma suppress effector T cell responses and associate with metastatic potential. Clin Cancer Res (2016) 22(16):4236–48. doi: 10.1158/1078-0432.CCR-15-2614 PMC4987192 27034329

[101] Zhang S Fujita H Mitsui H Yanofsky VR Fuentes-Duculan J Pettersen JS. Increased Tc22 and Treg/Cd8 ratio contribute to aggressive growth of transplant associated squamous cell carcinoma. PLoS One (2013) 8(5):e62154. doi: 10.1371/journal.pone.0062154 23667456 PMC3646982

[102] Clark RA Kupper TS. Il-15 and dermal fibroblasts induce proliferation of natural regulatory T cells isolated from human skin. Blood (2007) 109(1):194 –202. doi: 10.1182/blood-2006-02-002873 PMC1785078 16968902

[103] Yu P Fu YX. Tumor-infiltrating T lymphocytes: Friends or foes? Lab Invest (2006) 86(3):231–45. doi: 10.1038/labinvest.3700389 16446705

[104] Rutella S Lemoli RM. Regulatory T cells and tolerogenic dendritic cells: From basic biology to clinical applications. Immunol Lett (2004) 94(1-2):11–26. doi: 10.1016/j.imlet.2004.04.015 15234530

[105] Beyer M Schultze JL. Regulatory T cells in cancer. Blood (2006) 108(3):804–11. doi: 10.1182/blood-2006-02-002774 16861339

[106] Beyer M Kochanek M Giese T Endl E Weihrauch MR Knolle PA. *In vivo* peripheral expansion of naive Cd4+Cd25high Foxp3+ regulatory T cells in patients with multiple myeloma. Blood (2006) 107(10):3940–9. doi: 10.1182/blood-2005-09-3671 16410445

[107] Thornton AM Shevach EM. Cd4+Cd25+ immunoregulatory T cells suppress polyclonal T cell activation in vitro by inhibiting interleukin 2 production. J Exp Med (1998) 188(2):287–96. doi: 10.1084/jem.188.2.287 PMC2212461 9670041

[108] Ng WF Duggan PJ Ponchel F Matarese G Lombardi G Edwards AD. Human Cd4(+) Cd25(+) cells: A naturally occurring population of regulatory T cells. Blood (2001) 98(9):2736–44. doi: 10.1182/blood.v98.9.2736 11675346

[109] Bates GJ Fox SB Han C Leek RD Garcia JF Harris AL. Quantification of regulatory T cells enables the identification of high-risk breast cancer patients and those at risk of late relapse. J Clin Oncol (2006) 24(34):5373–80. doi: 10.1200/JCO.2006.05.9584 17135638

[110] Wolf D Wolf AM Rumpold H Fiegl H Zeimet AG Muller-Holzner E. The expression of the regulatory T cell-specific forkhead box transcription factor Foxp3 is associated with poor prognosis in ovarian cancer. Clin Cancer Res (2005) 11(23):8326–31. doi: 10.1158/1078-0432.CCR-05-1244 16322292

[111] Curiel TJ Coukos G Zou L Alvarez X Cheng P Mottram P. Specific recruitment of regulatory T cells in ovarian carcinoma fosters immune privilege and predicts reduced survival. Nat Med (2004) 10(9):942–9. doi: 10.1038/nm1093 15322536

[112] Frazzette N Khodadadi-Jamayran A Doudican N Santana A Felsen D Pavlick AC. Decreased cytotoxic T cells and tcr clonality in organ transplant recipients with squamous cell carcinoma. NPJ Precis Oncol (2020) 4:13. doi: 10.1038/s41698-020-0119-9 32550269 PMC7270180

[113] Zhang S Wu M Wang F. Immune regulation by Cd8(+) treg cells: Novel possibilities for anticancer immunotherapy. Cell Mol Immunol (2018) 15(9):805–7. doi: 10.1038/cmi.2018.170 PMC6203794 29503446

[114] Robb RJ Lineburg KE Kuns RD Wilson YA Raffelt NC Olver SD. Identification and expansion of highly suppressive Cd8(+)Foxp3(+) regulatory T cells after experimental allogeneic bone marrow transplantation. Blood (2012) 119(24):5898–908. doi: 10.1182/blood-2011-12-396119 22538855

[115] Freeman A Bridge JA Maruthayanar P Overgaard NH Jung JW Simpson F. Comparative immune phenotypic analysis of cutaneous squamous cell carcinoma and intraepidermal carcinoma in immune-competent individuals: Proportional representation of Cd8+ T-cells but not Foxp3+ regulatory T-cells is associated with disease stage. PLoS One (2014) 9(10):e110928. doi: 10.1371/journal.pone.0110928 25340823 PMC4207854

[116] Sedivy R Beck-Mannagetta J Haverkampf C Battistutti W Honigschnabl S. Expression of vascular endothelial growth factor-c correlates with the lymphatic microvessel density and the nodal status in oral squamous cell cancer. J Oral Pathol Med (2003) 32(8):455–60. doi: 10.1034/j.1600-0714.2003.00168.x 12901726

[117] Miyahara M Tanuma J Sugihara K Semba I. Tumor lymphangiogenesis correlates with lymph node metastasis and clinicopathologic parameters in oral squamous cell carcinoma. Cancer (2007) 110(6):1287–94. doi: 10.1002/cncr.22900 17674352

[118] Krediet JT Kanitakis J Bob A Schmitter J Krediet AC Rowert J. Prognostic value of the area and density of lymphatic vessels in cutaneous squamous cell carcinoma. J Dtsch Dermatol Ges (2016) 14(11):1114–21. doi: 10.1111/ddg.12880 27879093

[119] Karpanen T Egeblad M Karkkainen MJ Kubo H Yla-Herttuala S Jaattela M. Vascular endothelial growth factor c promotes tumor lymphangiogenesis and intralymphatic tumor growth. Cancer Res (2001) 61(5):1786–90. 11280723

[120] Neinaa YME El-Ashmawy AA Alshenawy HA Dorgham WL. The prognostic value of podoplanin expression in nonmelanoma skin cancers: Correlation with lymphatic vessel density. Am J Dermatopathol (2020) 42(6):432–8. doi: 10.1097/DAD.0000000000001561 31688008

[121] Kreppel M Krakowezki A Kreppel B Drebber U Wedemeyer I Mauch C. Podoplanin expression in cutaneous head and neck squamous cell carcinom prognostic value and clinicopathologic implications. J Surg Oncol (2013) 107(4):376–83. doi: 10.1002/jso.23238 22886751

[122] Hesse K Satzger I Schacht V Köther B Hillen U Klode J. Characterization of prognosis and invasion of cutaneous squamous cell carcinoma by podoplanin and e-cadherin expression. Dermatology (2016) 232(5):558–65. doi: 10.1159/000450920 27875814

[123] Cañueto J Cardeñoso-Álvarez E Cosano-Quero A Santos-Briz Á Fernández-López E Pérez-Losada J. The expression of podoplanin is associated with poor outcome in cutaneous squamous cell carcinoma. J Cutan Pathol (2017) 44(2):144–51. doi: 10.1111/cup.12859 27859466

[124] Wojciechowska-Zdrojowy M Szepietowski JC Matusiak Ł Dzięgiel P Puła B. Expression of podoplanin in non-melanoma skin cancers and actinic keratosis. Anticancer Res (2016) 36(4):1591–7. 27069135

[125] de Sousa SF Gleber-Netto FO de Oliveira-Neto HH Batista AC Nogueira Guimarães Abreu MH de Aguiar MC. Lymphangiogenesis and podoplanin expression in oral squamous cell carcinoma and the associated lymph nodes. Appl Immunohistochem Mol Morphol (2012) 20(6):588–94. doi: 10.1097/PAI.0b013e31824bb3ea 22495364

[126] Aiswarya A Suresh R Janardhanan M Savithri V Aravind T Mathew L. An immunohistochemical evaluation of podoplanin expression in oral leukoplakia and oral squamous cell carcinoma to explore its potential to be used as a predictor for malignant transformation. J Oral Maxillofac Pathol (2019) 23(1):159. doi: 10.4103/jomfp.JOMFP_272_17 31110440 PMC6503797

[127] Kim HY Rha KS Shim GA Kim JH Kim JM Huang SM. Podoplanin is involved in the prognosis of head and neck squamous cell carcinoma through interaction with vegf-c. Oncol Rep (2015) 34(2):833–42. doi: 10.3892/or.2015.4070 26081937

[128] Arimoto S Hasegawa T Takeda D Saito I Amano R Akashi M. Lymphangiogenesis and lymph node metastasis in oral squamous cell carcinoma. Anticancer Res (2018) 38(11):6157–62. doi: 10.21873/anticanres.12968 30396932

[129] Franses JW Baker AB Chitalia VC Edelman ER. Stromal endothelial cells directly influence cancer progression. Sci Transl Med (2011) 3(66):66ra5. doi: 10.1126/scitranslmed.3001542 PMC3076139 21248315

[130] Kakasheva-Mazhenkovska L Basheska N Crvenkova S Gordana P Milenkova L Janevska V. Correlation between microvessel density and morphological features in skin squamous cell carcinoma. Pril (Makedon Akad Nauk Umet Odd Med Nauki) (2017) 38(1):63–73. doi: 10.1515/prilozi-2017-0009 28593886

[131] Yamada S Ogasawara S Kaneko MK Kato Y. Lpmab-23: A cancer-specific monoclonal antibody against human podoplanin. Monoclon Antib Immunodiagn Immunother (2017) 36(2):72–6. doi: 10.1089/mab.2017.0001 28387591

[132] Mitsui H Suarez-Farinas M Gulati N Shah KR Cannizzaro MV Coats I. Gene expression profiling of the leading edge of cutaneous squamous cell carcinoma: Il-24-Driven mmp-7. J Invest Dermatol (2014) 134(5):1418–27. doi: 10.1038/jid.2013.494 PMC3989465 24270662

[133] Zaidi MR Merlino G. The two faces of interferon-Γ in cancer. Clin Cancer Res (2011) 17(19):6118–24. doi: 10.1158/1078-0432.CCR-11-0482 PMC3186825 21705455

[134] Lin WW Karin M. A cytokine-mediated link between innate immunity, inflammation, and cancer. J Clin Invest (2007) 117(5):1175–83. doi: 10.1172/JCI31537 PMC1857251 17476347

[135] Lewis AM Varghese S Xu H Alexander HR. Interleukin-1 and cancer progression: The emerging role of interleukin-1 receptor antagonist as a novel therapeutic agent in cancer treatment. J Transl Med (2006) 4:48. doi: 10.1186/1479-5876-4-48 17096856 PMC1660548

[136] Balkwill F. Tnf-alpha in promotion and progression of cancer. Cancer Metastasis Rev (2006) 25(3):409–16. doi: 10.1007/s10555-006-9005-3 16951987

[137] Yuan A Chen JJ Yao PL Yang PC. The role of interleukin-8 in cancer cells and microenvironment interaction. Front Biosci (2005) 10:853–65. doi: 10.2741/1579 15569594

[138] Yamada S Jinnin M Kajihara I Nakashima T Aoi J Harada M. Cytokine expression profiles in the sera of cutaneous squamous cell carcinoma patients. Drug Discovery Ther (2016) 10(3):172–6. doi: 10.5582/ddt.2016.01032 27169370

[139] Lathers DM Young MR. Increased aberrance of cytokine expression in plasma of patients with more advanced squamous cell carcinoma of the head and neck. Cytokine (2004) 25(5):220–8. doi: 10.1016/j.cyto.2003.11.005 15036248

[140] Skrinjar I Brailo V Vidovic-Juras D Vucicevic-Boras V Milenovic A. Evaluation of pretreatment serum interleukin-6 and tumour necrosis factor alpha as a potential biomarker for recurrence in patients with oral squamous cell carcinoma. Med Oral Patol Oral Cir Bucal (2015) 20(4):e402–7. doi: 10.4317/medoral.20373 PMC4523251 25858079

[141] Glick AB. The role of tgfβ signaling in squamous cell cancer: Lessons from mouse models. J Skin Cancer (2012) 2012:249063. doi: 10.1155/2012/249063 23326666 PMC3541634

[142] Smith CW Chen Z Dong G Loukinova E Pegram MY Nicholas-Figueroa L. The host environment promotes the development of primary and metastatic squamous cell carcinomas that constitutively express proinflammatory cytokines il-1alpha, il-6, gm-csf, and kc. Clin Exp Metastasis (1998) 16(7):655–64. doi: 10.1023/a:1006559811429 9932612

[143] Naganawa K Takayama E Adachi M Mitsudo K Iida M Kamiya-Mizuno M. Producing capabilities of interferon-gamma and interleukin-10 in peripheral blood from oral squamous cell carcinoma patients. Open Dent J (2015) 9:120–4. doi: 10.2174/1874210601509010120 PMC4397950 25893021

[144] Trikha M Corringham R Klein B Rossi JF. Targeted anti-Interleukin-6 monoclonal antibody therapy for cancer: A review of the rationale and clinical evidence. Clin Cancer Res (2003) 9(13):4653–65. PMC2929399 14581334

[145] Bachelot T Ray-Coquard I Menetrier-Caux C Rastkha M Duc A Blay JY. Prognostic value of serum levels of interleukin 6 and of serum and plasma levels of vascular endothelial growth factor in hormone-refractory metastatic breast cancer patients. Br J Cancer (2003) 88(11):1721–6. doi: 10.1038/sj.bjc.6600956 PMC2377148 12771987

[146] Grivennikov S Karin M. Autocrine il-6 signaling: A key event in tumorigenesis? Cancer Cell (2008) 13(1):7–9. doi: 10.1016/j.ccr.2007.12.020 18167335

[147] Lederle W Depner S Schnur S Obermueller E Catone N Just A. Il-6 promotes malignant growth of skin sccs by regulating a network of autocrine and paracrine cytokines. Int J Cancer (2011) 128(12):2803–14. doi: 10.1002/ijc.25621 20726000

[148] Ii M Yamamoto H Adachi Y Maruyama Y Shinomura Y. Role of matrix metalloproteinase-7 (Matrilysin) in human cancer invasion, apoptosis, growth, and angiogenesis. Exp Biol Med (Maywood) (2006) 231(1):20–7. doi: 10.1177/153537020623100103 16380641

[149] Mueller MM Peter W Mappes M Huelsen A Steinbauer H Boukamp P. Tumor progression of skin carcinoma cells in vivo promoted by clonal selection, mutagenesis, and autocrine growth regulation by granulocyte colony-stimulating factor and granulocyte-macrophage colony-stimulating factor. Am J Pathol (2001) 159(4):1567–79. doi: 10.1016/S0002-9440(10)62541-2 PMC1850484 11583982

[150] Mueller MM Fusenig NE. Constitutive expression of G-csf and gm-csf in human skin carcinoma cells with functional consequence for tumor progression. Int J Cancer (1999) 83(6):780–9. doi: 10.1002/(sici)1097-0215(19991210)83:6<780::aid-ijc14>3.0.co;2-c 10597195

[151] Mann EA Spiro JD Chen LL Kreutzer DL. Cytokine expression by head and neck squamous cell carcinomas. Am J Surg (1992) 164(6):567–73. doi: 10.1016/s0002-9610(05)80708-1 1463101

[152] Obermueller E Vosseler S Fusenig NE Mueller MM. Cooperative autocrine and paracrine functions of granulocyte colony-stimulating factor and granulocyte-macrophage colony-stimulating factor in the progression of skin carcinoma cells. Cancer Res (2004) 64(21):7801–12. doi: 10.1158/0008-5472.CAN-03-3301 15520186

[153] Wu F Weigel KJ Zhou H Wang XJ. Paradoxical roles of tgf-beta signaling in suppressing and promoting squamous cell carcinoma. Acta Biochim Biophys Sin (Shanghai) (2018) 50(1):98–105. doi: 10.1093/abbs/gmx127 29206939 PMC5846704

[154] Wolk K Witte E Witte K Warszawska K Sabat R. Biology of interleukin-22. Semin Immunopathol (2010) 32(1):17–31. doi: 10.1007/s00281-009-0188-x 20127093

[155] Res PC Piskin G de Boer OJ van der Loos CM Teeling P Bos JD. Overrepresentation of il-17a and il-22 producing Cd8 T cells in lesional skin suggests their involvement in the pathogenesis of psoriasis. PLoS One (2010) 5(11):e14108. doi: 10.1371/journal.pone.0014108 21124836 PMC2991333

[156] Chung Y Yang X Chang SH Ma L Tian Q Dong C. Expression and regulation of il-22 in the il-17-Producing Cd4+ T lymphocytes. Cell Res (2006) 16(11):902–7. doi: 10.1038/sj.cr.7310106 17088898

[157] Nograles KE Zaba LC Shemer A Fuentes-Duculan J Cardinale I Kikuchi T. Il-22-Producing "T22" T cells account for upregulated il-22 in atopic dermatitis despite reduced il-17-Producing Th17 T cells. J Allergy Clin Immunol (2009) 123(6):1244–52.e2. doi: 10.1016/j.jaci.2009.03.041 19439349 PMC2874584

[158] Abikhair M Mitsui H Yanofsky V Roudiani N Ovits C Bryan T. Cyclosporine a immunosuppression drives catastrophic squamous cell carcinoma through il-22. JCI Insight (2016) 1(8):e86434. doi: 10.1172/jci.insight.86434 27699266 PMC5033893

[159] Curd LM Favors SE Gregg RK. Pro-tumour activity of interleukin-22 in hpafii human pancreatic cancer cells. Clin Exp Immunol (2012) 168(2):192–9. doi: 10.1111/j.1365-2249.2012.04570.x PMC3390520 22471280

[160] Rangwala S Tsai KY. Roles of the immune system in skin cancer. Br J Dermatol (2011) 165(5):953–65. doi: 10.1111/j.1365-2133.2011.10507.x PMC3197980 21729024

[161] Clark RA Chong B Mirchandani N Brinster NK Yamanaka K Dowgiert RK. The vast majority of cla+ T cells are resident in normal skin. J Immunol (2006) 176(7):4431–9. doi: 10.4049/jimmunol.176.7.4431 16547281

[162] Mellman I Coukos G Dranoff G. Cancer immunotherapy comes of age. Nature (2011) 480(7378):480–9. doi: 10.1038/nature10673 PMC3967235 22193102

[163] Rosenberg SA. Cancer immunotherapy comes of age. Nat Clin Pract Oncol (2005) 2(3):115. doi: 10.1038/ncponc0101 16264884 PMC2656369

[164] Tamai H Watanabe S Zheng R Deguchi K Cohen PA Koski GK. Effective treatment of spontaneous metastases derived from a poorly immunogenic murine mammary carcinoma by combined dendritic-tumor hybrid vaccination and adoptive transfer of sensitized T cells. Clin Immunol (2008) 127(1):66–77. doi: 10.1016/j.clim.2007.12.001 18262845

[165] Topalian SL Weiner GJ Pardoll DM. Cancer immunotherapy comes of age. J Clin Oncol (2011) 29(36):4828–36. doi: 10.1200/JCO.2011.38.0899 PMC3255990 22042955

